# Fellow-Workers and Their Doings

**Published:** 1914-02-07

**Authors:** 


### ROUND THE WORLD.

### Fellow-Workers and their Doings.

[The Editor Invites Early Information and Contributions Marked "Fellow-Workers."]

Dr. George Graham, M.D., M.R.C.P., the newly appointed assistant physician to the East London Hospital for Children at Shadwell, is a Cambridge and "Bart.'s" man. He has also worked at Münich and at the West London Hospital. The year before last he was elected a Beit Memorial Fellow. Dr. Graham's researches have dealt with physiological chemistry, particularly in the matter of protein metabolism, on which he has written a good deal.

At the same time Mr. C. M. Kennedy, F.R.C.S., of the London Hospital, where he has lately been surgical registrar, has been appointed assistant surgeon to this well-known children's hospital. Mr. Kennedy has recently published important original observations on suppurative epiphysitis and gall-stone dyspepsia.

Dr. Richard Armstrong, the Curator of the Museum at St. Bartholomew's Hospital, is occupied in an interesting research in connection with lobar pneumonia, in the course of which he hopes to settle the question as to whether this disease is chiefly an air-borne or a hæmotogenous infection. Dr. Armstrong, who is a Lawrence Research Scholar at "Bart.'s," and had special opportunities of studying pneumonia when he was on the resident staff of the Great Ormond Street Hospital for Sick Children, is inclined to the belief that the pneumococci always reach the lungs through the air passages.

Dr. J. Hayward Willett, M.D., the new President of the North of England Obstetrical and Gynæcological Society, is a student and graduate of Liverpool University. As surgeon to the Hospital for Women and the Maternity Hospital at Liverpool, as well as in the office of examiner to the Manchester Centre of the Central Midwives Board, Mr. Willett has worked hard in the interests of his specialty. The new President of this important Obstetrical and Gynæcological Society, which was established in 1890, served as a civil surgeon in the South African War.

Mr. Frederick A. Anderson, M.D., D.P.H., who has been appointed assistant surgeon to the Ear, Eye, and Throat Hospital at Shrewsbury, is an old Dublin student. He was previously house surgeon to the Royal Victoria Eye and Ear Hospital, and is the inventor of a modified form of Stevenson's sclero-corneal trephine.

Everyone who attended the successful annual congress of the British Medical Association held at Brighton last summer will be gratified at the presentation just made to Dr. L. A. Parry by the members of the local division. Dr. Parry, who has been made the recipient of a fine case of cutlery, acted as honorary local secretary at the special meeting referred to, and its great success was largely due to his exertions. It is generally admitted that the Brighton gathering was one of the most successful that have been held during the past ten years. Dr. Parry is an M.D. Lond. and F.R.C.S. Eng., and holds the appointments of consulting surgeon to Hove Cripples' Guild, assistant surgeon to the Royal Alexandra Hospital for Sick Children, and medical officer to the Education Committee of the Hove Borough Council

Mr. Hastings Gilford, F.R.C.S., of Reading, who on Monday last, February 2, opened the annual course of lectures at the Royal College of Surgeons, is one of that praiseworthy band who have made time for scientific research, in spite of the claims of busy general practice. Mr. Gilford, who is an old Guy's man, has made many important observations on dwarfism and other problems of arrested development.

Dr. A. E. Garrod, M.D., F.R.C.P., whose researches on morbid arthritic conditions and metabolic disorders are well known, has lately been giving the alkaloid colchicine in place of the ordinary colchicum preparations in the treatment of acute gout. Of late there has been a movement in favour of giving pure alkaloids instead of the old-fashioned pharmacopœial remedies, but it has not always been found that simple active principles have had the same beneficial effect as the former treatment in various diseases.

The pioneer work of Sir Henry Howse, M.S., F.R.C.S., in introducing the methods of the late Lord Lister into London is perhaps not sufficiently appreciated. Writing to the *Lancet*, Dr. W. Hale White particularly emphasises the credit due to Sir Henry Howse, who evidently began a systematic trial of Listerism in the erysipelas wards at Guy's Hospital as early as 1871, and had, indeed, put into practice antiseptic principles in a small way when he was a house surgeon four years previously. The work and teachings of Sir Henry Howse are, of course, well known to all past and present students of "Guy's," where he was appointed full surgeon in 1875.

Dr. A. T. Nankivell, M.D., D.P.H., who has been appointed medical officer of health to the borough of Poole, had a brilliant career as a student at Guy's Hospital, where he distinguished himself in bacteriological and public health work. Dr. Nankivell was awarded the gold medal in the State Medicine branch at the M.D., London, examination in 1910, and has lately been medical officer of health at St. Austell.

Dr. R. McCombe, lately assistant school doctor for Margate, succeeds the late Dr. Bertram Thornton as medical officer of health for that borough. In his new office Dr. McCombe, who is an old Dublin student and an F.R.C.S.I., will combine the duties of medical officer of health, school medical officer, and police surgeon.

Dr. W. T. Rowe, of Nottingham, has lately been giving attention to the evidence of the shape and movements of the stomach in life that can be obtained by the use of bismuth meals and *x*-rays. Giving an account of his recent observations to the Nottingham Medico-Chirurgical Society on January 7, this investigator pointed out that his observations support the view that the stomach is a curved firm tube lying to the left hypochondrium, and not a flaccid sac resting haphazard in the upper part of the abdomen.

